# Functional analysis of a conserved site mutation in the DNA end processing enzyme PNKP leading to ataxia with oculomotor apraxia type 4 in humans

**DOI:** 10.1016/j.jbc.2023.104714

**Published:** 2023-04-13

**Authors:** Azharul Islam, Anirban Chakraborty, Stefano Gambardella, Rosa Campopiano, Altaf H. Sarker, Istvan Boldogh, Tapas Hazra

**Affiliations:** 1 Department of Internal Medicine, University of Texas Medical Branch, Galveston, Texas, USA; 2 IRCCS Neuromed & Department of Biomolecular Sciences, University of Urbino “Carlo Bo”, Urbino, Italy; 3 Life Sciences Division, Lawrence Berkeley National Laboratory, Berkeley, California, USA; 4 Department of Microbiology and Immunology, University of Texas Medical Branch, Galveston, Texas, USA

**Keywords:** PNKP, nuclear transport, DNA repair, neurodegeneration, AOA4

## Abstract

Polynucleotide kinase 3′-phosphatase (PNKP), an essential DNA end-processing enzyme in mammals with 3′-phosphatase and 5′-kinase activities, plays a pivotal role in multiple DNA repair pathways. Its functional deficiency has been etiologically linked to various neurological disorders. Recent reports have shown that mutation at a conserved glutamine (Gln) in PNKP leads to late-onset ataxia with oculomotor apraxia type 4 (AOA4) in humans and embryonic lethality in pigs. However, the molecular mechanism underlying such phenotypes remains elusive. Here, we report that the enzymatic activities of the mutant *versus* WT PNKP are comparable; however, cells expressing mutant PNKP and peripheral blood mononuclear cells (PBMCs) of AOA4 patients showed a significant amount of DNA double-strand break accumulation and consequent activation of the DNA damage response. Further investigation revealed that the nuclear localization of mutant PNKP is severely abrogated, and the mutant proteins remain primarily in the cytoplasm. Western blot analysis of AOA4 patient-derived PBMCs also revealed the presence of mutated PNKP predominantly in the cytoplasm. To understand the molecular determinants, we identified that mutation at a conserved Gln residue impedes the interaction of PNKP with importin alpha but not with importin beta, two highly conserved proteins that mediate the import of proteins from the cytoplasm into the nucleus. Collectively, our data suggest that the absence of PNKP in the nucleus leads to constant activation of the DNA damage response due to persistent accumulation of double-strand breaks in the mutant cells, triggering death of vulnerable brain cells—a potential cause of neurodegeneration in AOA4 patients.

Living organisms, throughout their lives, inevitably encounter various types of DNA damage, arising from exposure to endogenous reactive oxygen species and/or exogenous physical/chemical agents (1, 2). DNA strand breaks (SBs) with nonligatable DNA termini, such as 3′-phosphate and 5′-OH, which are formed directly due to DNA damaging agents or as repair intermediates are the most prevalent and deleterious forms of DNA damage (3, 4, 5, 6). Polynucleotide kinase 3′-phosphatase (PNKP) is a bifunctional DNA end-processing enzyme that possesses both 3′-phosphatase and 5′-kinase activities (3, 7, 8, 9, 10) and thus can generate the canonical 3′-OH and 5′-phosphate termini, respectively, to facilitate the gap filling and ligation essential for the repair of DNA SBs. PNKP is thus involved in multiple DNA repair pathways, such as base excision repair, single-strand break, and double-strand break (DSB) repair *via* the nonhomologous end-joining pathway (6, 11, 12, 13, 14, 15). Apart from its nuclear role, we and others have provided evidence for PNKP’s critical role in mitochondrial base excision repair (14, 16). Several studies revealed that three distinct neurological disorders are predominantly associated with mutations in PNKP (13): (i) microcephaly with early-onset seizures, a neurodevelopmental disease associated with microcephaly (17); (ii) ataxia with oculomotor apraxia type 4 (AOA4), which exhibits progressive cerebellar atrophy and ataxia oculomotor apraxia (18, 19, 20) although some individuals possess both microcephaly and progressive cerebellar atrophy (20, 21, 22, 23, 24); (iii) Charcot-Marie-Tooth disease 2B2, associated with mild axonal peripheral polyneuropathy and relatively late-onset cerebellar ataxia (25, 26, 27). Mutation of PNKP also causes a defect in cortical development (17, 28). PNKP inactivation in mouse neural progenitor cells results in neurodevelopmental abnormalities and postnatal death (28). Therefore, deficient PNKP activity has been linked to neurological/developmental disorders, and the persistent DNA SBs are postulated to lead to the degeneration of selected neuron and glial cell populations, a common and critical feature of many acute and chronic neurological diseases. Notably, our studies have provided evidence of abrogated PNKP activity leading to subsequent impairment of single-strand break repair and classical nonhomologous end-joining pathway in neurodegenerative diseases, such as spinocerebellar ataxia type 3 and Huntington’s disease where polyglutamine repeat expansion is attributed as the genetic basis of the disease (3, 29, 30, 31).

Structural studies have revealed that PNKP has three domains: an N-terminal fork head-associated (FHA) domain (residues 6–110) which is linked to two catalytic domains, a phosphatase (residues 146–337), and a kinase domain (residues 341–516) *via* a linker region (residues 111–145) (7, 32, 33). The FHA is a regulatory domain, which is critical for the recruitment of PNKP to DNA damage sites *via* its interaction with other repair proteins, such as XRCC1 (34, 35, 36) or XRCC4 (5, 15, 37, 38). Additionally, the FHA domain may also play a role in the nuclear localization of PNKP. Recently, the nuclear localization signal (NLS) of PNKP has been identified, and the critical residues of NLS were determined to be lysine 138, arginine 139, and arginine 141, all of which are located in the linker region (39). Studies have also revealed that arginine 35, located in the FHA domain, has additive effects on nuclear localization and nuclear distribution of PNKP (39). The FHA domain is present in a large range of proteins, and FHA domain-containing proteins are involved in diverse cellular processes including DNA repair (40, 41, 42). Sequence alignment analyses of the FHA domains show the presence of an evolutionarily conserved histidine in the majority of FHA domain-containing proteins, including the DNA repair proteins, Nijmegen breakage syndrome 1, and Aprataxin and polynucleotide kinase/phosphatase-like factor (43, 44). Interestingly, unlike other FHA domain-containing proteins, PNKP and Aprataxin have an evolutionarily conserved glutamine instead of histidine across lower to higher species. However, the biological implication of this specific but highly conserved mutation has not been studied extensively. Recently, a homozygous mutation of PNKP p.Gln50Glu (p.Q50E) was reported in an Italian AOA4 patient (45). Another report demonstrated embryonic lethality in pigs due to a missense mutation at a conserved Gln (p.Gln96Arg; p.Q96R) in PNKP involving the same residue (46). Therefore, we have mutated the evolutionarily conserved glutamine (residue 50) to glutamic acid and arginine, individually, and investigated the biological implications by assessing the functional properties of mutant PNKP in mammalian cells. Our study revealed that the enzymatic activities were not significantly altered; however, nuclear localization of the PNKP mutants (both p.Q50E and p.Q50R) is severely abrogated in human embryonic kidney (HEK293) cell lines and the peripheral blood mononuclear cells (PBMCs) of Italian AOA4 patient, leading to sustained activation of the DNA damage response (DDR). We thus postulate that persistent accumulation of DNA DSBs is a potential cause for AOA4 pathologies in humans and/or embryonic lethality in pigs.

## Results

### Mutations at Q50 in PNKP had minimal impact on its 3′-phosphatase or 5′ kinase activity

**Figure 1:**
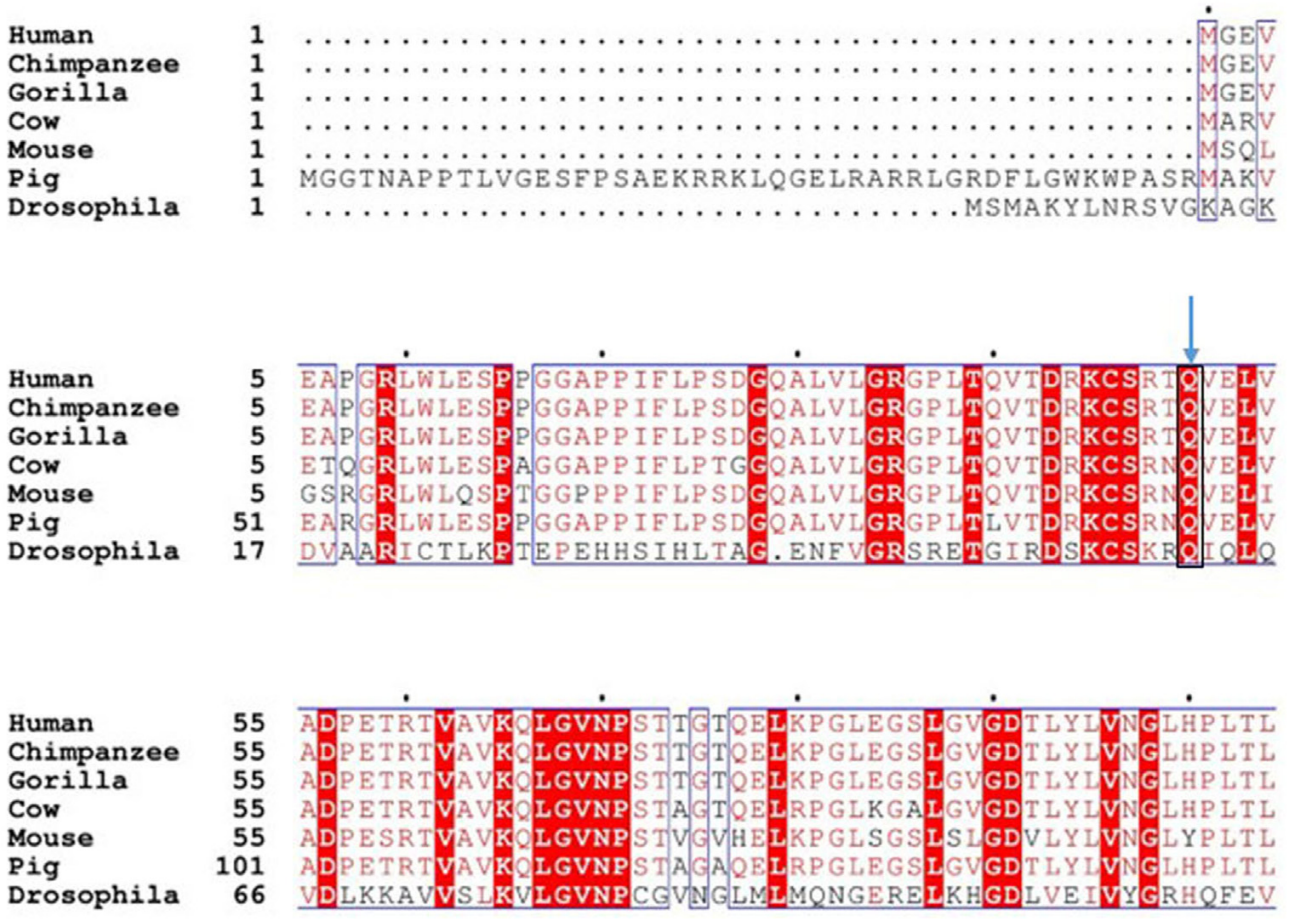
**Multiple sequence alignment of PNKP protein across different species.** PNKP, polynucleotide kinase 3′-phosphatase.

**Figure 2:**
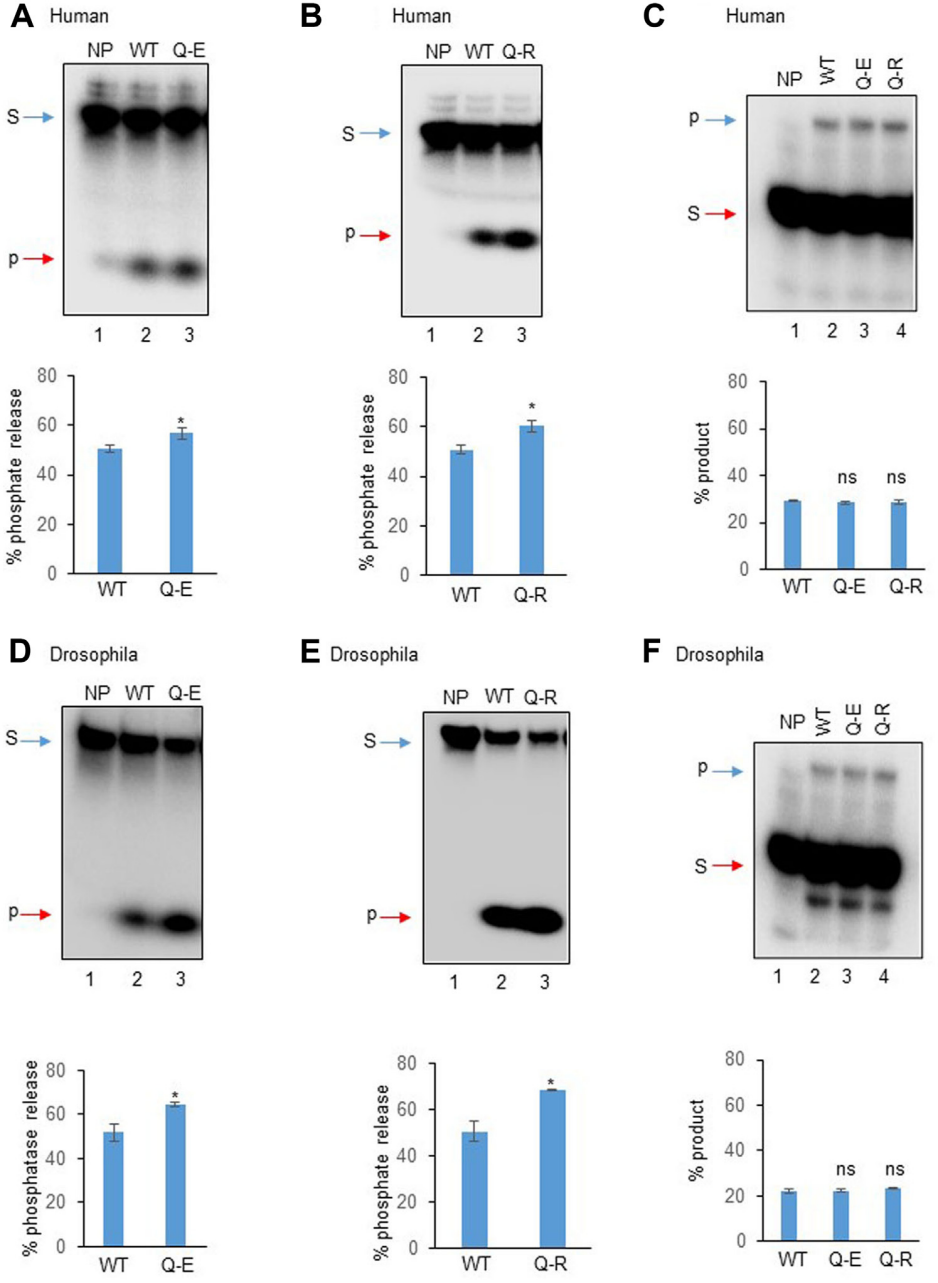
**Effects of p.Q50E and p.Q50R mutations on PNKP’s activity.***A* and *B*, *upper panel*, 3′-phosphatase assay of purified mutant human PNKP (p.Q50E or p.Q50R) (lane 3) *versus* WT (lane 2). Lane 1: no protein (NP), substrate only. *D* and *E*, similar 3′-phosphatase assay of Drosophila mutant (lane 3) *versus* WT (lane 2) PNKP. *Lower panels*, quantitation of the products (% of released phosphate) is represented in the bar diagram (n = 3, ∗*p* < 0.05 between WT *versus* mutants). The 5′-kinase activity of the purified mutant human (*C*) or Drosophila (*F*) PNKP (lanes 3 and 4) *versus* corresponding WT (lane 2). Lane 1: no protein (NP), substrate only. *Lower panels*, quantitation of the products (% phosphorylated product) is represented in the bar diagram (n = 3, ns = nonsignificant, *p* > 0.05). In each case, S: substrate and P: products. PNKP, polynucleotide kinase 3′-phosphatase.

Two recent reports have shown a homozygous mutation at conserved glutamine (Q-50 in humans and Q-96 in pigs) in the FHA domain of PNKP leading to AOA4 in an Italian patient (p.Q50E) and embryonic lethality in pigs (p.Q96R) (45, 46). To determine the functional significance of the conserved glutamine residue, we performed the amino acid sequence alignment of PNKP from lower to higher species using the ClustalW 2.1 multiple sequence alignment tool. Sequence alignment showed the conserved nature of the glutamine residue in *Drosophila melanogaster*, mouse, pig, gorilla, chimpanzee, cows, and humans (Fig. 1*A*). To determine the effect of p.Q50E and p.Q50R mutations on the biological function of human PNKP, we analyzed PNKP’s 3′-phosphatase and 5′-kinase activities using recombinant proteins. We found that both mutants (p.Q50E or p.Q50R) seemed to show a slight increase in the 3′-phosphatase activity (Fig. 2, *A* and *B*) but observed no change in the 5′-kinase activity (Fig. 2*C*), when compared to WT PNKP. Similarly, we investigated the effect of these mutations on the activity of *Drosophila* PNKP (29) and could not detect any significant alteration, consistent with our findings with human PNKP (Fig. 2, *D*–*F*). Overall, our findings indicate that a single mutation in the FHA domain does not have a significant impact on the enzymatic activities of PNKP.

### PNKP mutation led to SB accumulation in nuclear DNA but not in the mitochondrial DNA

**Figure 3:**
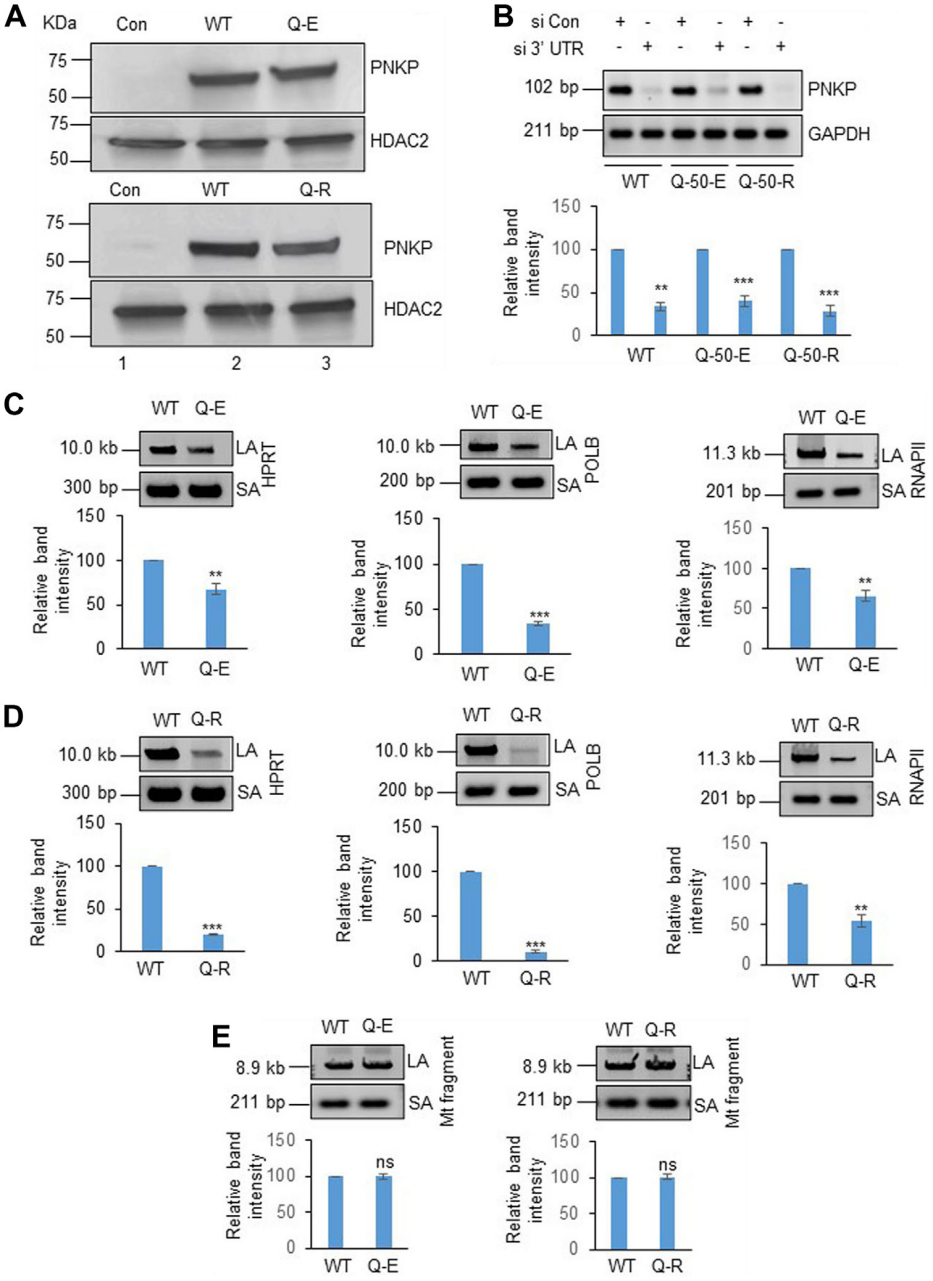
**Long amplicon PCR to detect the DNA strand breaks in mutant cells compared to WT**. *A*, Western blots with anti-FLAG Ab to show the levels of PNKP in the whole cell extracts of p.Q50E (*upper panel*) or p.Q50R (*lower panel*) expressing stable cell lines (lane 3) compared to WT PNKP-expressing cell line (lane 2). Vector-expressing HEK293 cells were used as a control (lane 1). HDAC2: used as a loading control. *B*, *upper panel*, the representative agarose gel shows the extent of depletion of endogenous PNKP in WT and p.Q50E- or p.Q50R-expressing stable cell lines by 3′UTR-specific siRNA. *Lower panel*, the bar diagram represents the relative expression level of endogenous PNKP normalized with the expression of endogenous control GAPDH and presented graphically as normalized relative band intensity with the control siRNA-transfected samples considered as 100 arbitrarily (n = 3, ∗∗*p* < 0.01, ∗∗∗*p* < 0.005). *C* and *D*, *upper panels*, representative agarose gel images of amplification of a long fragment of HPRT (10.4 kb), POLB (12.2 kb), and RNAP II (11.3 kb) and a small fragment of the corresponding genes from p.Q50E- (*C*) or p.Q50R- (*D*) expressing cells compared to WT PNKP-expressing cells as described above. *Lower panels*, the bar diagrams represent the normalized (with short PCR amplicon) relative band intensity with the WT-PNKP–expressing sample in each case arbitrarily set as 100 (n = 3, ∗∗*p* < 0.01, ∗∗∗*p* < 0.005). *E*, *upper* and *lower panels*, similar LA-qPCR involving long mitochondrial DNA fragment (8.9 kb) normalized with a short amplicon from the cell lines described above and the corresponding bar diagrams representing normalized band intensity (n = 3, ns = *p* > 0.05). HDAC2, histone deacetylases 2; HEK cell lines, human embryonic kidney cell lines; HPRT, hypoxanthine-guanine phosphoribosyltransferase; LA-qPCR, long amplicon quantitative PCR; PNKP, polynucleotide kinase 3′-phosphatase; RNAP II, RNA polymerase II; SBs, strand breaks.

PNKP is an essential DNA end-processing enzyme. To understand the deleterious effect of such conserved site mutations in PNKP, we generated stable cell lines ectopically expressing FLAG-tagged WT and mutant (p.Q50E or p.Q50R) PNKP. We selected the clones that express a similar level of WT or mutant of PNKP (Fig. 3*A*). To investigate the effect of the mutant PNKP, we depleted endogenous PNKP using 3′UTR-specific siRNA in these stable cell lines (Fig. 3*B*) and analyzed DNA damage accumulation in three representative genes (hypoxanthine-guanine phosphoribosyltransferase [HPRT], polymerase beta [POLB], and RNA polymerase II [RNAP II]) by long amplicon quantitative PCR (LA-qPCR). Our results demonstrated a significant increase in DNA SB accumulation in the p.Q50E or p.Q50R mutant-expressing cells, individually, compared to WT (Fig. 3, *C* and *D*). On the contrary, DNA SB accumulation was not observed in the mitochondrial genomic DNA in stable cell lines expressing p.Q50E or p.Q50R mutants compared to WT (Fig. 3*E*). Thus, we conclude that these mutations in PNKP lead to functional deficiency in repair of DNA SBs, specifically within the nucleus.

### Mutations (p.Q50E or p.Q50R) in PNKP impede its nuclear localization

**Figure 4:**
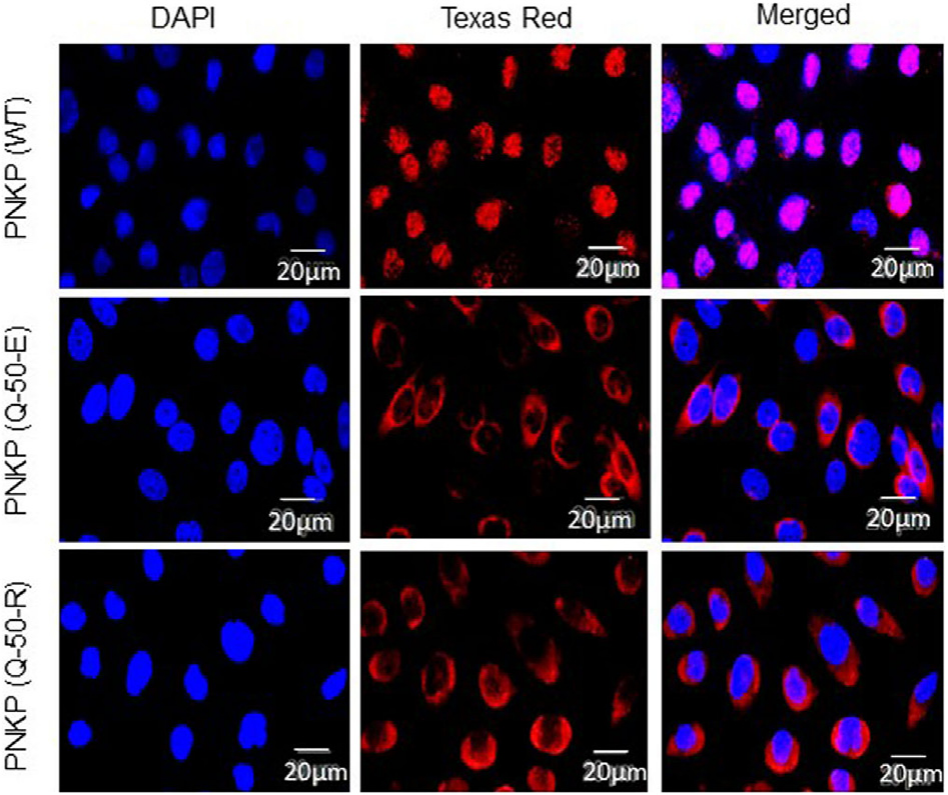
**Microscopic imaging to assess the subcellular localization of WT *versus* mutant PNKP.** Stable cell lines expressing WT and mutant PNKP (p.Q50E or p.Q50R) were fixed, permeabilized, and stained with an anti-FLAG Ab. Nuclei were counterstained with DAPI. Microscopic images were acquired individually using *blue* (461 nm) fluorescent for DAPI staining (*left panels*) and *red* (594 nm) fluorescent for Ab staining (*middle panel*). Images of the two colors were merged (*right panel*), and pictures were taken in a 20 μm area as shown in the figure. DAPI, 4′,6-diamidino-2-phenylindole; PNKP, polynucleotide kinase 3′-phosphatase.

Despite the comparable level of enzymatic activities in WT *versus* mutants of PNKP, we were surprised to observe that both mutant cells accumulated significantly higher levels of DNA SBs in the nuclear but not the mitochondrial genome. This observation led us to investigate the subcellular distribution of WT and mutant PNKP in the stable cell lines. Immunohistochemical analysis of the individual cell line using anti-FLAG Ab showed that the nuclear localization of mutant (p.Q50E or p.Q50R) PNKP was severely abrogated. We found that both mutants are predominantly localized in the cytoplasm, while WT PNKP was observed mostly in the nucleus (Fig. 4). Repair of 3′-phosphate containing DNA DSBs requires the presence of PNKP in the nucleus. We therefore conclude that the mislocalization of mutant PNKP in the cytoplasm is the major cause of DNA damage accumulation in the nucleus.

### Nuclear transport of PNKP is abrogated by p.Q50E or p.Q50R mutations, but mitochondrial localization remains unaltered in stable cell lines

**Figure 5:**
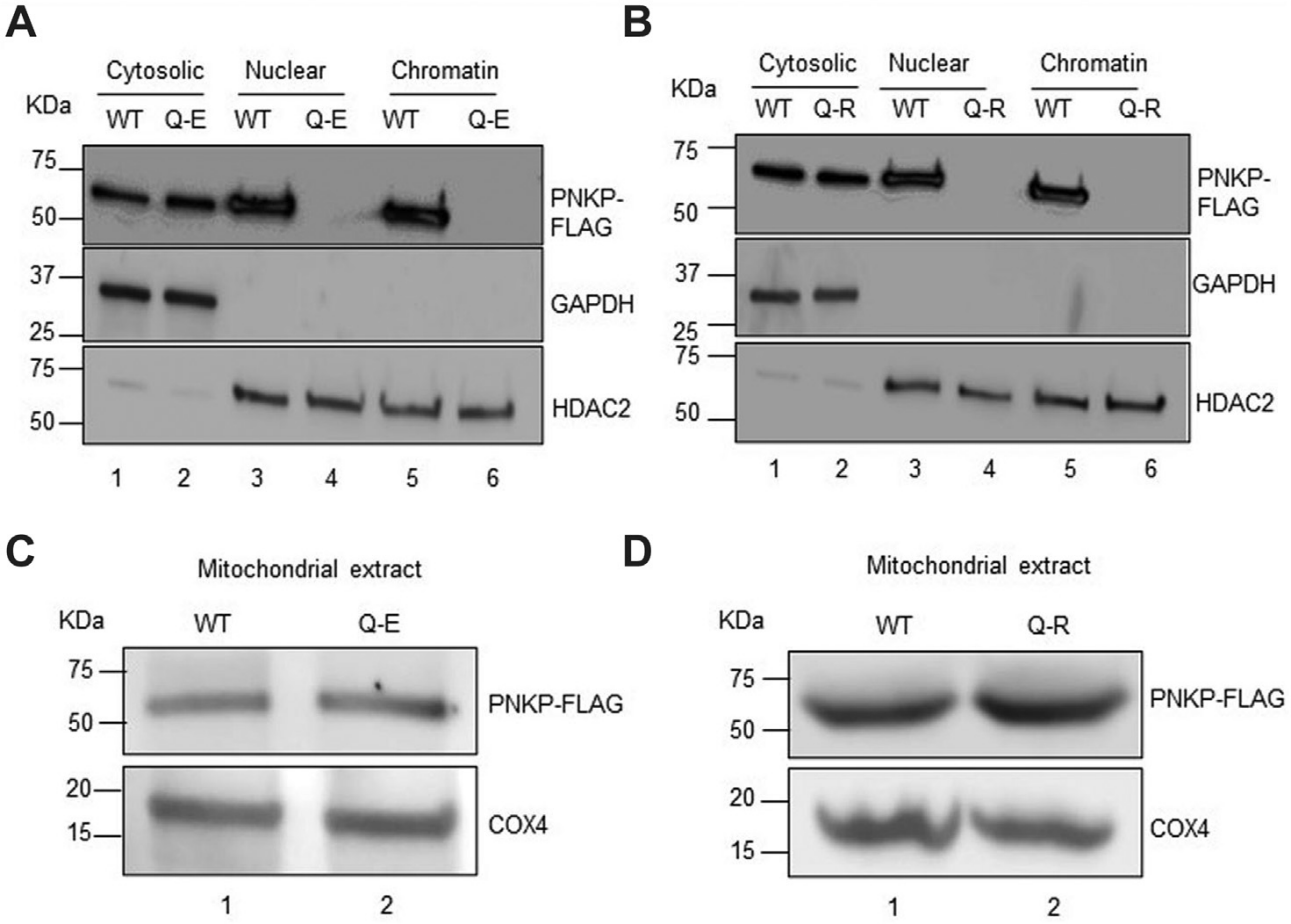
**Subcellular distribution of WT and mutant PNKP in the stable cell lines.** Western blots with anti-FLAG Ab to show the localization of WT *versus* mutant p.Q50E (*A*) or p.Q50R (*B*) PNKP in cytosolic (lane 1 and 2), nuclear (lanes 3 and 4), and chromatin fractions (lanes 5 and 6) prepared from the stable cell lines. GAPDH and HDAC2 are used as cytosolic and nuclear/chromatin extract purity as well as loading controls, respectively. Similarly, the mitochondrial localization of mutant p.Q50E (*C*) or p.Q50R (*D*) PNKP (lane 2) was determined as compared to their WT counterparts (lane 1) by Western blotting. COX4: used as a mitochondrial loading control. COX4, cytochrome c oxidase subunit 4; HDAC2, histone deacetylases 2; PNKP, polynucleotide kinase 3′-phosphatase.

To validate the results of microscopic imaging, we prepared cytoplasmic, nuclear, chromatin, and mitochondrial extracts from the stable cell lines expressing both WT and mutant (p.Q50E or p.Q50R) PNKP. Initially, we assessed the purity of these fractions by the presence or absence of appropriate marker proteins and then determined the subcellular localization of WT *versus* mutant PNKP in the fractions by Western blot analysis using an anti-FLAG antibody. Results showed that the level of both WT and mutant PNKP was very much comparable in the cytoplasm. However, the nuclear level of mutant PNKP (p.Q50E or p.Q50R) was severely abrogated compared to WT (Fig. 5, *A* and *B*). In contrast, the Western blot analysis showed that mutations (p.Q50E or p.Q50R) in PNKP did not interfere with its mitochondrial localization (Fig. 5, *C* and *D*). These results are consistent with the LA-qPCR data that showed impaired DNA repair in the nucleus, whereas mitochondrial DNA repair was unaltered in these stable cell lines.

### p.Q50E or p.Q50R mutations in PNKP impede its interaction with the nuclear pore complex protein importin alpha but not with importin beta

**Figure 6:**
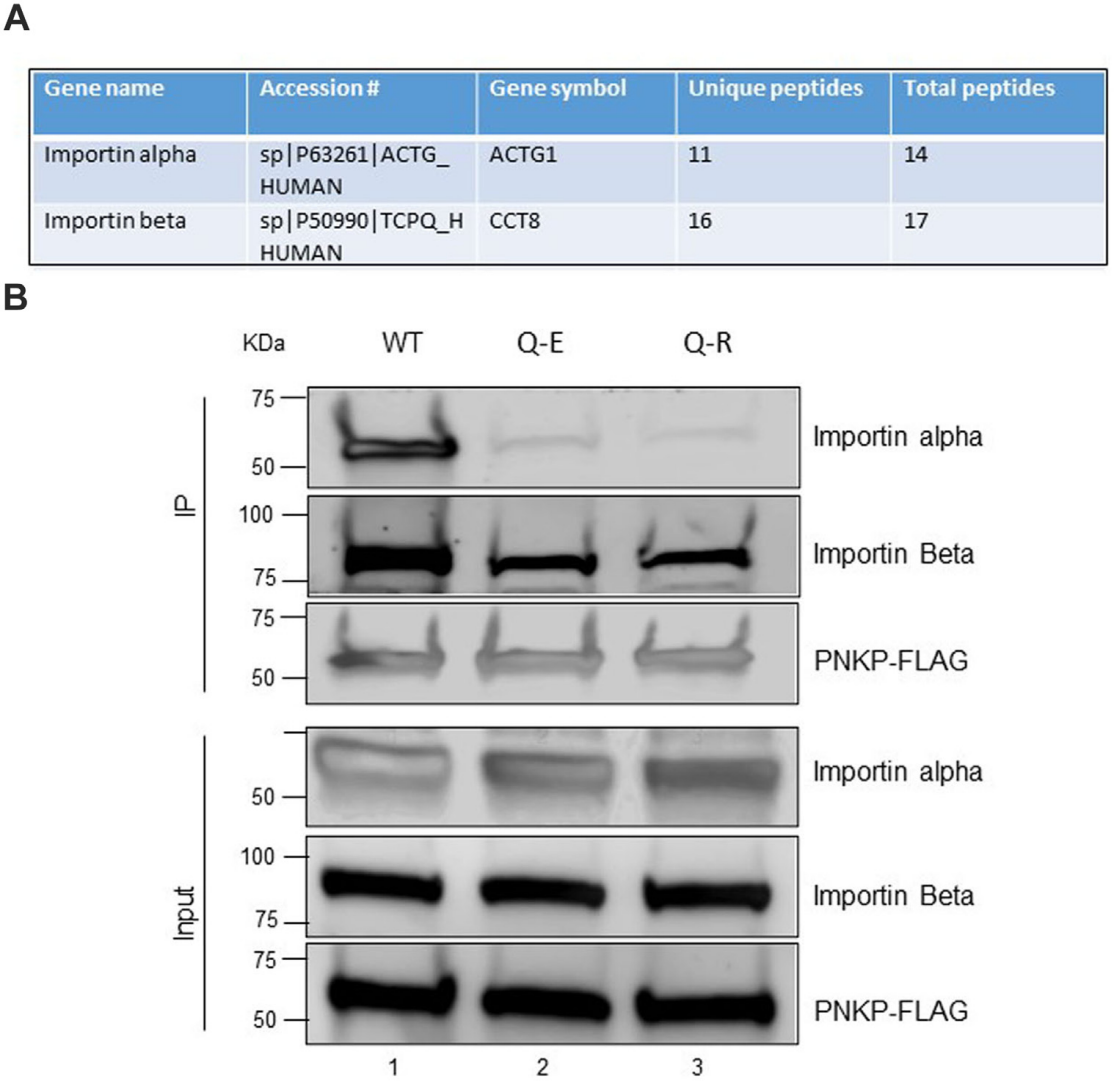
**Interaction of WT and mutant (p.Q50E or p.Q50R) PNKP with nuclear pore complex proteins (importin alpha and importin beta)**. *A*, the data of tandem mass spectrometry analysis of PNKP immunocomplex demonstrated the association of WT PNKP with importin alpha and importin beta as represented by total *versus* unique peptide scores. *B*, co-IP analysis of WT (lane 1), p.Q50E (lane 2), and p.Q50R (lane 3) PNKP was performed in the FLAG peptide–eluted complex from nuclear extracts prepared from the corresponding stable cell lines using anti-FLAG antibody and probed with importin alpha and importin beta. The same blot was probed with anti-FLAG Ab to show an equal amount of the eluted immunoprecipitated product (*upper panels*). The input nuclear extracts used for co-IP were run as controls, and the blot was probed with the anti-importin alpha, anti-importin beta, and anti-FLAG antibodies individually (*lower panels*). PNKP, polynucleotide kinase 3′-phosphatase.

Microscopic imaging and immunoblot analyses showed the abrogation of PNKP’s localization into the nucleus and consequently, the inhibition of recruitment to the chromatin due to p.Q50E or p.Q50R mutations. Therefore, to understand the mechanism of exclusion of PNKP from the nucleus, we explored the interaction of PNKP with the nuclear pore complex proteins, importin alpha and importin beta—two major proteins that perform an indispensable role in ferrying proteins from cytoplasm to the nucleus—by mass spectrometry and co-immunoprecipitation (co-IP) experiments. Mass spectrometry data indicated the presence of importin alpha and importin beta in the WT–PNKP immunocomplex (Fig. 6*A*). To further confirm the findings of mass spectrometry, we performed co-IP experiments from cell lines expressing WT and mutant PNKP. Western blot analysis clearly showed that the association of mutant (p.Q50E or p.Q50R) PNKP with importin alpha was severely abrogated but the association with importin beta was not (Fig. 6*B*). These results clearly suggest a mechanism of WT PNKP’s nuclear translocation in mammalian cells *via* its interaction with importin alpha and importin beta and also explain the mechanistic basis of abrogated nuclear import of the mutant PNKP.

### The abrogation of PNKP’s nuclear localization and subsequent DNA damage accumulation is evident in the PBMCs of Italian AOA4 patient

**Figure 7:**
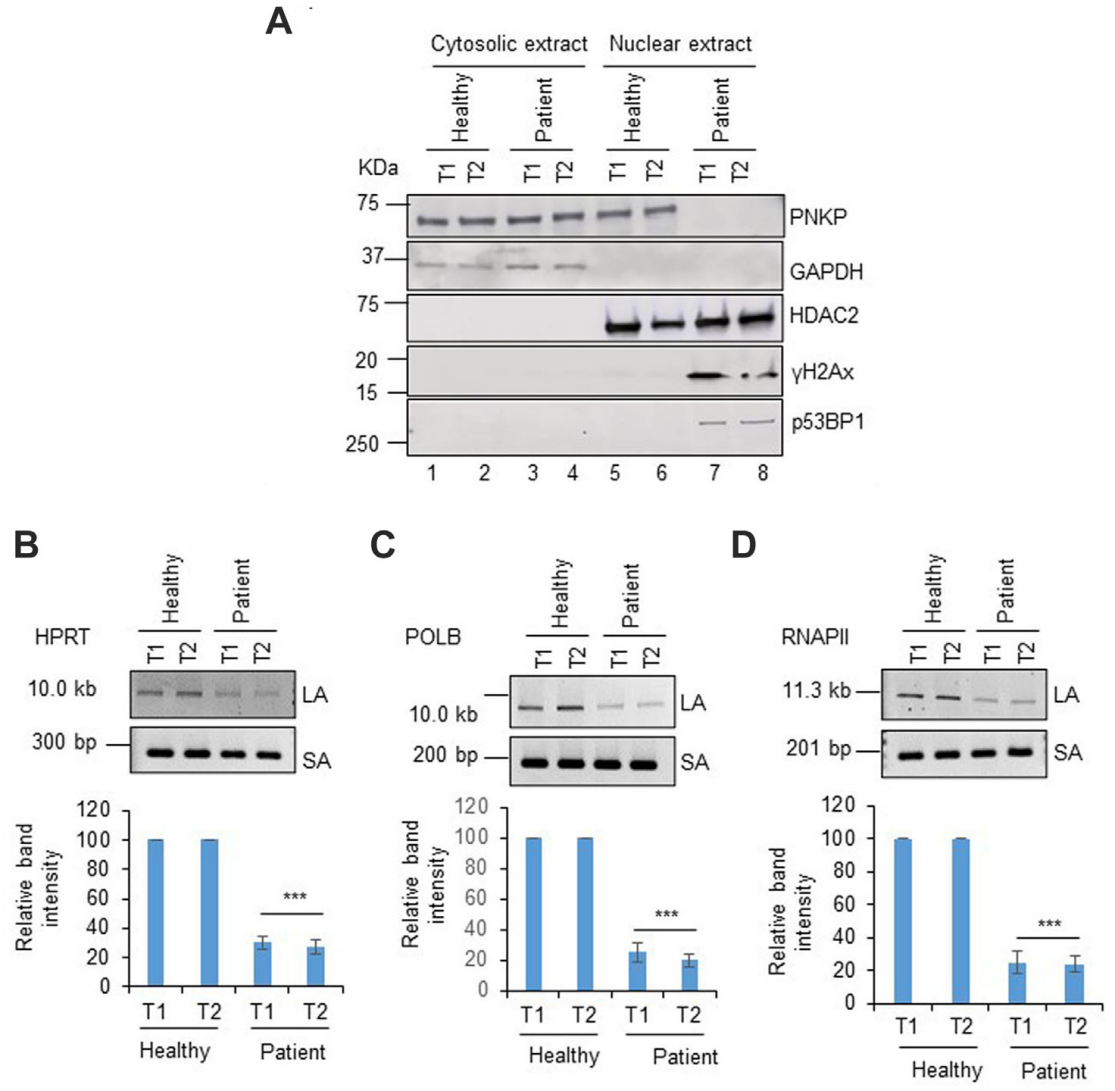
**Subcellular localization of PNKP and analysis of DNA SBs in the PBMCs of Italian AOA4 patient and their healthy sibling.***A*, Western blot was performed in the cytosolic (lanes 1–4) and nuclear fractions (lanes 5–8) of PBMCs of AOA4 patient (lanes 3–4, 7–8) *versus* healthy sibling (lanes 1–2, 5–6) and probed with the Abs against the proteins indicated *right* of each panel. GAPDH and HDAC2 are used as cytosolic and nuclear loading controls. *B*–*D*, *upper panels*, representative agarose gel images of amplification of a long fragment of HPRT (10.4 kb), POLB (12.2 kb), and RNAP II (11.3 kb) and a small fragment of the corresponding genes from a healthy sibling and AOA4 patients as described above. *Lower panels*, the bar diagrams represent the normalized (with short PCR amplicon) relative band intensity with the normalized band intensity for the healthy sibling arbitrarily set as 100 (n = 3, ∗∗∗*p* < 0.005). T1 and T2 represent replicate samples collected at two different time points. AOA4, ataxia with oculomotor apraxia type 4; HDAC2, histone deacetylases 2; HPRT, hypoxanthine-guanine phosphoribosyltransferase; PBMC, peripheral blood mononuclear cells; PNKP, polynucleotide kinase 3′-phosphatase; POLB, polymerase beta; RNAP II, RNA polymerase II; SBs, strand breaks.

Finally, to confirm the cell culture data, we investigated PNKP’s nuclear translocation and DNA SB accumulation in the PBMCs of an AOA4 patient carrying a homozygous p.Q50E mutation. Subcellular expression analyses of PNKP in the PBMCs of an Italian AOA4 patient and his healthy sibling showed findings similar to that observed in HEK293 stable cell lines expressing WT and mutant (p.Q50E) PNKP. Although a similar level of PNKP expression was observed in the cytoplasmic fraction, the translocation of PNKP was severely abrogated into the nucleus of the AOA4 patient compared to his healthy sibling (Fig. 7*A*). Importantly, we observed a significant increase in the level of γH2AX and p53BP1 (both DSB markers) in the nuclear extracts of AOA4 patient PBMCs, indicating chronic activation of DDR proteins. To explore the status of DNA SB, long amplicon PCR was performed using genomic DNA extracted from the PBMCs of the AOA4 patient and his healthy sibling as control. As speculated, LA-PCR analysis of three representative genes (HPRT, POLB, and RNAP II) showed a significant amount of DNA damage accumulation in the nuclear genome (Fig. 7, *B*–*D*). Collectively, our data demonstrate that defects in DNA repair in the nuclear genome due to the impairment of mutant PNKP’s nuclear translocation is a plausible cause for the onset of AOA4 pathology.

## Discussion

A spectrum of neurological diseases, although rare, such as microcephaly with early-onset seizures, progressive cerebellar atrophy and polyneuropathy, and AOA4 are associated with mutations in different domains of PNKP (47). Among those, all AOA4-related mutations in PNKP are mostly located in the kinase domain (18, 45). Variants in the FHA are mainly reported in individuals who manifest only seizures without neurodegeneration or microcephaly. In contrast, p.Q50E is the only variant in the FHA domain that recently has been reported as responsible for AOA4 in an Italian patient (19, 45). Notably, a similar conserved site missense mutation (p.Q96R) caused embryonic lethality in pigs (46). Therefore, we investigated the mechanistic basis for the association of the mutant with such severe pathological conditions. For this purpose, we characterized the relevant mutants biochemically *in vitro* and in cells and found that the 3′-phosphatase and 5′-kinase activities of recombinant p.Q50E or p.Q50R are very much comparable to WT PNKP. Surprisingly, however, the cells expressing mutant PNKP (both p.Q50E and p.Q50R) accumulated a significant amount of DNA SBs in the nuclear but not in the mitochondrial genome. Thus, our results reiterate that mutant forms of PNKP are deficient in their nuclear function. Our subsequent studies have demonstrated that the mutants are predominantly present in the cytoplasm but not in the nucleus or chromatin. Based on this observation, we conclude that the accumulation of DNA damage is due to the abrogation of nuclear localization of the PNKP mutants. We then further identified that the mutation in PNKP affected its transport *via* nuclear pores due to the failure of an interaction between mutant (p.Q50E or p.Q50R) PNKP with importin alpha, without hindering its interaction with importin beta. These experimental findings have demonstrated the importance of the interaction of PNKP with nuclear pore protein importin alpha for its translocation to the nucleus. A vast majority of NLS sequences are recognized by importin alpha and help translocate the protein into the nucleus through the nuclear pore complex *via* an interaction with importin beta; however, a significant number of proteins can be transported into the nucleus passively without NLS *via* direct interaction with importin beta, such as ribosomal proteins (48) and the viral proteins HIV-1, Tat, and Rev (49). In addition, many proteins interact with importin alpha *via* the non-NLS–mediated interfaces as their noncargo roles for translocation into the nucleus (50, 51). Therefore, we postulate that mutation at Q50 results in the failure of PNKP’s NLS recognition/interaction with the nuclear pore protein (importin alpha). Although the NLS (residues 133–142) of PNKP has already been identified (39), multiple accessory factors (NLS-dependent or NLS-independent) are likely to affect the nuclear import of PNKP. An in-depth mechanistic assessment of these various possibilities has remained beyond the scope of the present study.

We observed a widespread accumulation of DNA damage in the mutant PNKP-expressing cells, due to the lack of entry of mutant PNKP into the nucleus. Under these conditions, the cells mimic the physiology similar to *pnkp*-null status. PNKP’s critical role during development is well established as the global knockout of *pnkp* gene is embryonic lethal (28). Thus, it is significant that p.Q96R mutation harboring pigs display embryonic lethality as we expect a similar abrogation of nuclear localization of PNKP. Additionally, Shimada *et al.* has elegantly shown that the loss of *pnkp* greatly diminished neurogenesis and oligodendrogenesis due to the accumulation of persistent DNA SBs (28). Due to high energy demand, the metabolic activity is relatively higher in brain cells, which can lead to the persistent generation of reactive oxygen species and be further aggravated under pathogenic conditions inducing DNA damage, including DNA DSBs. Since PNKP is an essential end-processing enzyme, the absence of PNKP in the nucleus will give rise to sustained accumulation of unrepaired strand breaks leading to persistent activation of DDR pathways. This can render a subpopulation of neuronal cells vulnerable to apoptosis, leading to neurodegenerative features as seen in AOA4 patients.

The manifestation of the vast majority of diseases associated with PNKP is reflected in the blood, and thus, all cell types in the blood of AOA4 patient should carry the p.Q50E mutation. AOA4 is a relatively rare genetic disease and thus, the availability of postmortem patient brain tissue of affected regions is an extremely remote possibility. Additionally, due to the easy availability of blood samples together with the complex procedure of isolating human brain tissues from living individuals as well as some institutional regulatory barriers, we have analyzed the nuclear localization of PNKP and subsequent DNA damage in the PBMCs of the Italian AOA4 patient and compared to a healthy individual. Nonetheless, the nuclear translocation is indeed severely abrogated, and a significantly higher DNA SB accumulation is observed in the PBMCs of the AOA4 patient compared to the healthy individual. We also observed an elevated level of DDR proteins, such as γH2AX and p53BP1. This finding demonstrates that the onset of AOA4 pathology is strongly linked to the failure of PNKP’s repair activity of the nuclear genome and subsequent chronic activation of DDR pathways. Future studies should be undertaken to identify disease-causing mutations, such as Q50, in the population, which may provide a window into developing patient-specific, mechanism-based therapies.

## Experimental procedures

### Cell lines, expression plasmids, and antibodies

HEK293 cells and HEK293-derived stable cell lines expressing WT and mutant PNKP-FLAG were cultured and maintained in Dulbecco's modified Eagle's medium (F12-50/50) (Cellgro) containing 10% fetal bovine serum (R & D Systems-Bio-techne brand) and antibiotics (penicillin, streptomycin, and amphotericin B) (Thermo Fisher Scientific) under 5% CO_2_ and 95% relative humidity at 37 °C. All the cell lines (initial source: ATCC) were authenticated by short tandem repeat analysis in the University of Texas Medical Branch (UTMB) Molecular Genomics Core. We routinely tested our experimental cells using the Mycoalert Mycoplasma Detection Kit (Lonza) according to the manufacturer’s protocol, and the cells were found to be free from mycoplasma contamination. Mammalian expression plasmid, pCDNA3 was purchased from Thermo Fisher Scientific, and bacterial expression plasmids, pET24a(+) and pET28a(+), were procured from Novagen/Millipore Sigma. The following primary antibodies were used in this study: monoclonal anti-FLAG M2 (Millipore Sigma), polyclonal anti-PNKP (BioBharati), rabbit polyclonal γH2AX and p53BP1 (Cell Signaling Technology). The remaining antibodies (rabbit polyclonal anti-GAPDH, HDAC2, Cox4, importin alpha, importin beta) were purchased from GeneTex. The dilution of all primary antibodies was 1:500 except GAPDH (1:5000). The dilution of the secondary horseradish peroxidase (HRP)–conjugated anti-rabbit IgG was 1:5000 and the anti-mouse IgG was 1:2000. All antibodies were diluted in 5% skimmed milk.

### Cloning of human and drosophila PNKP into mammalian and bacterial expression vectors

Human PNKP (gene accession # NM_007254.4) with a FLAG tag was cloned into pCDNA3 between *Hin*dIII and *Bam*HI sites as described earlier (52) to generate the plasmid pCDNA3-PNKP-FLAG. The coding DNA sequenceof human PNKP was further amplified from a pCDNA3-PNKP-FLAG by the Q5 hot start high-fidelity DNA polymerase (New England Biolabs). Similarly, *D. melanogaster* PNKP (gene accession # NM_141535.4) was amplified from pACUH-dPNKP, a kind gift from Dr Xu Chen at the University of California San Diego (UCSD). The primers used to amplify human and *drosophila* PNKP are listed in Table 1. Amplified PCR product of human PNKP was cloned into the *Nde*I-*Bam*HI sites of pET28A(+), and the *Drosophila* PNKP fragment was inserted into *Eco*RI-*Sal*I sites of pET24A(+) with an N-terminal 6X Histidine tag in frame. All PNKP clones were confirmed by sequencing in the UTMB Molecular Genomics core.

**Table 1:** Primers used in the study

Primers	Gene	Nucleotide sequence 5′-3′	Purpose
F1	hPNKP	TAAGCACATATGGGCGAGGTGGAGGCC	For cloning human PNKP in pET28A(+)
R1	hPNKP	TAAGCAGGATCC GCCCTCGGAGAACTGGCAG	For cloning human PNKP in pET28A(+)
F2	dPNKP	TAAGCAGAATTCATGT CTATGGCTAAATACC	For cloning Drosophila PNKP in pET24A(+)
R2	dPNKP	TTTCTCGACCAAGTAC	For cloning Drosophila PNKP in pET24A(+)
F3	hPNKP	CTCCAGAACTgaaGTGGAGCTGG	For introducing Q-50-E mutation in human PNKP
R3	hPNKP	CACTTCCGGTCCGTAACC	For introducing Q-50-E mutation in human PNKP
F4	hPNKP	CTCCAGAACTcgaGTGGAGCTGG	For introducing Q-50-R mutation in human PNKP
R4	hPNKP	CACTTCCGGTCCGTAACC	For introducing Q-50-R mutation in human PNKP
F5	dPNKP	CTCCAGAACTgagGTGGAGCTGG	For introducing Q-50-E mutation in *Drosophila* PNKP
R5	dPNKP	CACTTCCGGTCCGTAACC	For introducing Q-50-E mutation in *Drosophila* PNKP
F6	dPNKP	CTCCAGAACTcggGTGGAGCTGG	For introducing Q-50-R mutation in *Drosophila* PNKP
R6	dPNKP	CACTTCCGGTCCGTAACC	For introducing Q-50-R mutation in *Drosophila* PNKP
F7	HPRT	TGGGATTACACGTGTGAACCAACC	LA-qPCR
R7	HPRT	GCTCTACCCTCTCCTCTACCGTCC	LA-qPCR
F8	HPRT	TGCTCGAGATGTGATGAAGG	SA-PCR
R8	HPRT	CTGCATTGTTTTGCCAGTGT	SA-PCR
F9	POLB	CATGTCACCACTGGACTCTGCAC	LA-qPCR
R9	POLB	CCTGGAGTAGGAACAAAAATTGCT	LA-qPCR
F10	POLB	AGTGGGCTGGATGTAACCTG	SA-PCR
R10	POLB	CCAGTAGATGTGCTGCCAGA	SA-PCR
F11	RNAPOLII	AGGGGGTGTGTGAAGCAAAA	LA-qPCR
R11	RNAPOLII	AGGGAGGCACATCTCTAGCA	LA-qPCR
F12	RNAPOLII	CGCATTGACTTGCGTTTCCA	SA-PCR
R12	RNAPOLII	CTGGGCAGCAACAGCCTTTA	SA-PCR
F13	mtFRAGMENT	TTTCATCATGCGGAGATGTTGGATGG	LA-qPCR
R13	mtFRAGMENT	TCTAAGCCTCCTTATTCGAGCCGA	LA-qPCR
F14	mtFRAGMENT	CCCCACAAACCCCATTACTAAACCCA	SA-PCR
R14	mtFRAGMENT	TTTCATCATGCGGAGATGTTGGATGG	SA-PCR
F15	GAPDH	CATCACTGCCACCCAGAAGA	Internal control for gene expression
R15	GAPDH	TTCTAGACGGCAGGTCAGGT	Internal control for gene expression
F16	3′-UTR-PNKP	GAGATCCCGTTCCGGCTATG	Expression of PNKP-3′-UTR
R16	3′-UTR-PNKP	CAGCGTTTATTGTGGAGGGG	Expression of PNKP-3′-UTR
F17	hPNKP	CTGGCACCCCTCTGGTGT	Gene sequencing for confirmation of human PNKP cloning
R17	hPNKP	CAGCCGGGGTTTGACTTC	Gene sequencing for confirmation of human PNKP cloning
F18	dPNKP	TAA TAC GAC TCA CTA TAG GGG	Gene sequencing for confirmation of *Drosophila* PNKP cloning
R18		GCT AGT TAT TGC TCA GCG G	Gene sequencing for confirmation of *Drosophila* PNKP cloning

### Site-directed mutagenesis

A missense mutation was introduced into human PNKP by replacing the glutamine 50 (CAA) with glutamic acid (GAA) and arginine (CGA), individually, using the Q5 site-directed mutagenesis kit (New England Biolabs) according to the manufacturer’s protocol. PCR primers were designed using NEBaseChanger for generating mutations. Similarly, a missense mutation was introduced into the PNKP of *D. melanogaster* by replacing glutamine 61 (CAG) with glutamic acid (GAG) and arginine (CGG), individually. The primers used to introduce these mutations are listed in Table 1. The introduced mutations were confirmed by sequencing in UTMB Molecular Genomics Core.

### Expression and purification of human and drosophila PNKP and *in vitro* phosphatase and kinase assay

Expression and purification of both human and drosophila PNKP were performed following the protocol mentioned earlier (29, 52, 53) with minor modifications. Both human and drosophila PNKP-expressing vectors were transformed into BL21-codon plus RIPL *Escherichia coli* competent cells (Thermo Fisher Scientific), and the colonies were inoculated and grown in LB broth at 37 °C until the absorbance reached 0.4 to 0.6. Induction of PNKP was performed with 0.5 mM IPTG and allowed expression overnight at 16 °C in an incubator shaker. PNKP was purified by immobilized metal-affinity chromatography using HisPur Cobalt Resin (Thermo Fisher Scientific) according to the manufacturer’s protocol. Immobilized metal-affinity chromatography–purified PNKP was further purified by passing through the HiTrap SP cation exchange column (GE HealthCare) using FPLC (AKTA Pure; GE HealthCare). FPLC column–purified PNKP was run in SDS-PAGE and then stained with Coomassie brilliant blue, and the concentration was determined in comparison with the known concentrations of bovine serum albumin (BSA). The 3′-phosphatase activity of PNKP was determined by the *in vitro* 3′ phosphatase assay as described (6, 16, 29) with minor modification. Briefly, 3′-phosphate–containing substrate (5 pmol) labeled with γP^32^ ATP (6) was incubated individually with WT and mutant PNKP at 37 °C for 13 min in phosphatase assay buffer (25 mM Tris–HCl pH 7.5, 100 mM NaCl, 5 mM MgCl_2_, 1 mM DTT, 10% glycerol, and 0.1 μg/μl acetylated BSA). Alongside, the 5′-kinase assay of purified PNKP was performed as described (13, 54) with minor modifications. γP^32^-labeled ATP was incubated in kinase assay buffer (25 mM Tris–HCl pH 7.5, 130 mM KCl, 10 mM MgCl_2_, 50 nM ATP, 1 mM DTT, 2.5% glycerol) along with 1.0 μg/μl acetylated BSA, 0.6 μl labeled substrate for 1 h at 30 °C. For the phosphatase assay, 2 ng purified PNKP and 7.5 pmol cold substrate were used, and for the kinase assay, 100 fmol of PNKP and 2.5 pmol cold substrate were used. The radioactive bands were visualized *via* PhosphorImager (GE HealthCare) and quantitated using ImageQuant software (https://www.cytivalifesciences.com/en/us/shop/protein-analysis/molecular-imaging-for-proteins/imaging-software/imagequant-tl-10-2-analysis-software-p-28619). The data were represented as % product released from the radiolabeled substrate.

### Generation of PNKP-FLAG–expressing stable cell lines

Individual clones of WT or mutant PNKP-FLAG in pCDNA3 were used to make PNKP-FLAG–expressing stable cell lines by transfecting these clones into HEK293 cell lines. Briefly, 1 μg of PNKP-expressing vector was transfected into HEK293 cells in a 6-well plate using lipofectamine 2000 (Invitrogen) as per manufacturer’s protocols, and clones were selected with 400 mM of geneticin (G418 sulfate, Millipore Sigma) starting 48 h post-transfection. Surviving cells (in which PNKP-FLAG–expressing plasmids were stably integrated) were propagated, and the expression of the PNKP-FLAG in stable cell lines was confirmed by immunoblotting using an anti-FLAG antibody.

### Mass spectrometry analysis for detection of WT-PNKP–associated protein(s) by LC-MS/MS

FLAG-tagged WT PNKP was IP’d from the nuclear extract of stable cell lines using FLAG beads (SIGMA). Following IP, the beads were washed three times with the buffer (20 mM Hepes pH 7.9, 0.5 mM EDTA, 0.25% Triton X-100, 250 mM KCl, 10% glycerol) to remove nonspecifically-bound proteins and eluted with two bed volume of elution buffer (20 mM Tris–HCl pH 7.5, 150 mM NaCl, 10% glycerol) containing 150 μg/ml FLAG peptide (SIGMA). Thirty microliters of the eluted IP’d complex was then separated by SDS-PAGE. A Coomassie brilliant blue stained/destained gel slice of PNKP-associated proteins was excised into approximately 1 mm^3^ pieces. Gel pieces were subjected to mass spectrometry analysis in the Taplin Biological Spectrometry facility at Harvard Medical School as described (55, 56). Peptide sequences (and hence protein identity) were determined by matching protein or translated nucleotide databases with the acquired fragmentation pattern by the software program, Sequest (https://www.selectscience.net/products/sequest-cluster/?prodID=10319) (Thermo Finnigan) (57). All databases include a reversed version of all the sequences, and the data were filtered between a one and two percent peptide false discovery rate. The results of mass spectrometry were further validated by performing a similar IP experiment followed by Western analyses of the FLAG-eluted product for the presence of relevant proteins (importin alpha and importin beta) in the PNKP–FLAG immunocomplex.

### Collection of blood and isolation of PBMCs

Blood samples were collected from one female caucasian healthy subject (35 years old) and one patient affected by late-onset AOA4 (MIM616267). The patient is a 60-year-old Italian male, onset at 54, with cerebellar dysarthria, gait ataxia, mild bilateral dysmetria, oculomotor apraxia, distal weakness of the arms and legs, distal hypoesthesia, and absent tendon reflexes. He is a carrier of homozygous PNKP mutation rs756746191:C>G [NP_009185.2:p.(Gln50Glu); NM_007254.3:c.(148C>G)], represented as p.Q50E in this manuscript. According to the American College of Medical Genetics and Genomics (ACMG) guidelines, this is classified as a likely pathogenic variant (45). The samples were processed by gradient separation with Ficoll (Lymphoprep) according to the manufacturer’s instructions and stored at −80 °C for future downstream processing. This study was approved by the ethics committee (code number CGM-01) and registered in clinicaltrials.gov (NCT03084224). Replicate samples were collected at two different times and they are presented as sample T1 and T2 in this manuscript.

### Extraction of cytoplasmic, nuclear, and chromatin fractions and Western blotting

Cytoplasmic, nuclear, and chromatin extracts were prepared following the procedure described earlier (31, 52, 58) with little modification. Benzonase (0.030 units/μl) was added to the chromatin extraction buffer and incubated at 37 °C for 45 min. The same protocol was utilized for the preparation of cytoplasmic and nuclear extract from the PBMCs of an Italian AOA4 patient and his healthy sibling. Cytoplasmic, nuclear, and chromatin extract (20 μg) were separated by SDS-PAGE, and Western blot analysis was performed using anti-FLAG/PNKP/γH2AX/p53BP1 antibodies, as described earlier (52, 53, 59).

### Indirect immunofluorescence microscopic imaging

WT and mutant PNKP- (p.Q50E or p.Q50R) expressing HEK293 cells were plated on collagen pretreated cover glass (Roche Applied Sciences). Cells at 80% confluence were fixed with acetone-methanol (1:1) for 20 min at room temperature and dried. At use, cells were rinsed and permeabilized using 0.1% (w/v) Triton-X-100 diluted in PBS (PBS with Tween 20 [PBST]) for 5 min, incubated with 1% BSA for 1 h at room temperature, and then blocked with human FcR blocking reagent (Miltenyi Biotech). Anti-DDDK-tag–specific Ab (FLAG, Cell Signaling) was added at a dilution (1:300) in PBST determined in preliminary studies and incubated for 1 h at 37 °C. After washing the cells three times in PBST, a secondary antibody conjugated to Alexa Fluor 594 (goat anti-mouse) was added and incubated for 1 h at 37 °C. After washing in PBST (three times), cells were dried and mounted with VECTASHIELD/4′6-diamidino-2-phenylindole hydrochloride (Vector Laboratories). Over 30 randomly selected fields of view per sample were photographed using a WHN10×/22 eyepiece and a 60× objective (field of view is 1.1 mm and camera correction is 0.63) on an Olympus BX53-P polarizing microscope.

### Total RNA extraction, complementary DNA preparation, and Reverse transcription quantitative polymerase chain reaction (RT-qPCR)

Stable cell lines expressing WT and mutant (p.Q50E or p.Q50R) PNKP-FLAG were transfected with either control or PNKP 3′UTR-specific siRNA (Horizon Discovery) in a 6-well plate using Lipofectamine 2000 reagent as per manufacturer’s protocol. The sequences of PNKP’s 3′UTR-specific siRNA were as follows: sense sequence: CCU CCA CAA UAA ACG CUG U UU and antisense sequence: ACA GCG UUU AUU GUG GAG G UU. Forty-eight hours post-transfection, cells were harvested, and total RNA was extracted using TRIzol reagent (Thermo Fisher Scientific) using manufacturer's protocol. One microgram of total RNA was used to make complementary DNA (cDNA) using the prime script RT reagent kit (Takara Bio Inc), according to the manufacturer’s protocol, and RT-PCR was performed using 1 μl of complementary DNA to amplify the 3′UTR region of the endogenous PNKP gene as well as the housekeeping gene (GAPDH) using quick-load Taq 2x master mix (New England Biolabs). The primers for amplifying the 3′-UTR region of PNKP and GAPDH are listed in Table 1. The amplified PCR products were run in agarose gel and stained with ethidium bromide, and the image was captured by Gel Doc EZ imager (Bio-Rad). The quantitation of bands was done using ImageJ software (https://imagej.net/software/imagej/) and the expression of endogenous PNKP with 3′-UTR was normalized by the GAPDH. The data were represented as relative expression levels with the expression of endogenous PNKP in control siRNA-transfected cells considered as 100.

### Long amplicon quantitative PCR

Aforementioned siRNA-transfected stable cells were harvested at 48 h post-transfection, and genomic DNA was extracted using the QIAamp DNA Micro kit (Qiagen) according to the manufacturer’s protocol and the concentration was determined by the NanoVue (GE HealthCare). Gene-specific LA-qPCR assays for measuring DNA SBs were performed as described earlier (29, 52, 60) using LongAmp Taq DNA Polymerase (New England BioLabs). Genomic DNA was used as a template to amplify a 10.4 kb region of the HPRT gene, 12.2 kb of the DNA POLB gene, and 11.3 kb of the RNAP II gene using the primers described previously (3, 59), (Table 1). Initial optimization of the assays was performed to assure the linearity of PCR amplification with regard to the number of cycles and genomic DNA concentration (10–20 ng). Since the amplification of a small region would be independent of DNA damage, a small DNA fragment (short amplicon PCR) from the same gene was also amplified for normalization of the amplified large fragment. The LA-qPCR and short amplicon PCR reactions were set for all three genes from the same stock of diluted genomic DNA samples, to overcome the variations in PCR amplification during sample preparation. The amplified products were then stained with ethidium bromide and visualized on agarose gels followed by quantitation with ImageJ software. The extent of DNA damage was calculated based on the relative quantification of the ratio of the large fragment and the short fragment. Extraction of genomic DNA from the Italian AOA4 patient and his healthy sibling was also performed using the QIAamp DNA Micro kit (Qiagen) according to the manufacturer’s protocol, and all subsequent steps were the same as described for the stable cell lines.

### Statistical analysis

A two-sided unpaired Student’s *t* test (http://www.ruf.rice.edu/∼bioslabs/tools/stats/ttest.html) was used for the analysis of statistical significance between two sets of data. Significance was evaluated at level *p* > 0.05 (ns), *p* < 0.05 (∗), *p* < 0.01 (∗∗) and *p* < 0.005 or 0.0001 (∗∗∗), as the case may be.

## Data availability

The data that support the findings of this study are available from the corresponding author upon request.

## Conflict of interest

The authors declare that they do not have any conflicts of interests.
